# Cavitation Erosion Characteristics of the EN AW-6082 Aluminum Alloy by TIG Surface Remelting

**DOI:** 10.3390/ma16072563

**Published:** 2023-03-23

**Authors:** Ion Mitelea, Ilare Bordeașu, Florin Frant, Ion-Dragoș Uțu, Corneliu Marius Crăciunescu, Cristian Ghera

**Affiliations:** 1 Department of Materials and Fabrication Engineering, Politehnica University Timisoara, Bulevardul Mihai Viteazul nr.1, 300222 Timisoara, Romania; 2 Department of Mechanical Machines, Equipment and Transports, Politehnica University Timisoara, Bulevardul Mihai Viteazul nr.1, 300222 Timisoara, Romania

**Keywords:** TIG remelting, aluminum alloy, cavitation erosion

## Abstract

Components made of aluminum alloys operating under cavitation erosion conditions have low performance and therefore a reduced lifetime. The degradation of these components is a consequence of the repetitive implosion of cavitation bubbles adjacent to the solid surface. In this paper, the effect of the rapid re-melting and solidification modification of the surface microstructure of parts of an Al-based alloy strengthened by artificial ageing on the reduction of material loss through cavitation erosion was investigated. The heat source used was the electric arc generated between a tungsten electrode and the workpiece (i.e., TIG). Local surface melting was performed at different values of linear energy (El = 6600–15840 J/cm), varying the current between 100 A and 200 A, at a constant voltage of 10 V. The obtained results showed an increase in the surface microhardness at values of 129–137 HV0.05 and a decrease in the erosion rate from 0.50 µm/min, characteristic of the artificial ageing heat treatment, to 0.10–0.32 µm/min, specific to TIG re-melted layers. For the study of the cavitational erosion mechanism, investigations were carried out by optical microscopy and scanning electron microscopy. The results showed that the improvement of the cavitational erosion resistance by surface melting was a consequence of the increase in microstructural homogeneity and grains refinement.

## 1. Introduction

Degradation of component surfaces by cavitation erosion is a result of the rapid formation, growth, and collapse of bubbles in liquids due to strong pressure fluctuations [1]. Cavitation erosion involves different types of interaction between metallurgical, chemical, mechanical and hydrostatic processes [2,3]. This phenomenon causes surface damage and loss of material due to the growth and collapse of bubbles, which are due to local pressure fluctuations within the liquid flowing around the surface of the parts [1,4]. In detail, when there is a localized pressure drop, the liquid reaches the vapor pressure level and undergoes a phase change, forming bubbles (cavities) containing vapor. Cavitation bubbles last only until the low-pressure zone is left. When the fluid returns to the quiet zone, the bubbles immediately implode, forming a shock wave that strikes and erodes the surface of the part it is in contact with.

A series of research papers analyzed the behavior of various types of metallic materials exposed to cavitation during their operation [5,6,7,8,9]. It is well known that among technical alloys, those with an aluminum base have the lowest cavitation erosion resistance [2,4]. In surface engineering, to reduce cavitation wear, various methods are used to prepare coatings applied to a substrate, such as the welding deposition method, thermal spraying, chemical or physical vapor deposition, etc. [1,6]. Li et al. [10] showed that the application of a thermochemical nitriding treatment improved the cavitation erosion behavior of some Ti-based non-ferrous alloys, which can be explained by the generation of a homogeneous microstructure with a single layer of α-Ti(N) in the diffusion nitrogen-enriched zone. Kwok et al. [11] and Zhang et al. [12] highlighted the advantages offered by the laser technique in the deposition of protective layers against the cavitation erosion of engineering materials.

Man et al. [13] used a laser technique for SiC/Si_3_N_4_ surface alloying of the AA6061 aluminum alloy. They found that the cavitation erosion resistance was improved three times compared to that of the reference material, while there was no significant improvement in the 100% SiC-alloyed specimen.

However, these surface strengthening methods have some disadvantages, as they result in the presence of pores, relatively low adhesion, inhomogeneous dilution, and a non-uniform microstructure [14,15,16,17], which limit the applications in cavitation environments. For these reasons, modifying the surface properties without changing the chemical composition is an innovative approach to avoid the mentioned defects.

Due to the high cost of laser beam operations and the need for vacuum in electron beam equipment, attention has been focused on using TIG welders that are cheap, flexible and easy to handle for surface modification by local melting. The TIG electric arc produces enough thermal energy to perform some surface treatments; it offers some significant advantages, including selective hardening, minimal distortion of the components, controllable depth of the modified layer, and absence of filler material.

The purpose of this paper was to investigate the effect of local TIG surface remelting of a 6xxx series aluminum base alloy—which could be strengthened by aging—in terms of structural transformation mechanism, onto the cavitation erosion resistance. The 6xxx series aluminum base alloys are frequently used for the production of components that undergo severe cavitation erosion phenomena during service, such as diesel cylinder liners, pistons, pumps, hydrofoils, valves, sluice gates, combustion chambers, etc. [1,2,4,6,9,13,18].

## 2. Experimental Procedure

The material used in the research, type EN AW-6082, (EN AW-AlSi1MgMn according to EN 573) was delivered in the form of sheets with the following dimensions: length L = 300 mm; width l = 150 mm, and thickness g = 30 mm.

The nominal chemical composition of the alloy was: Si = 1.18%, Fe = 0.39%, Cu = 0.065%, Mn = 0.70%, Mg = 1.32%, Cr = 0.10%, Ni = 0.015%, Zn = 0.044%, Ti = 0.011%, Ga = 0.01%, V = 0.023%, Al = Rest%.

**Figure 1:**
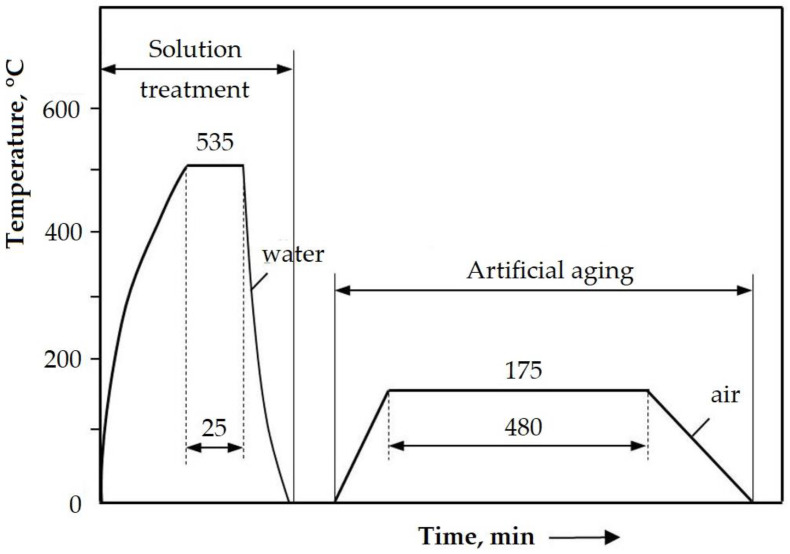
Heat treatment cycle.

The heat treatment was carried out by applying a solution at 535 ± 5 °C/25 min/water, followed by artificial aging at 175 ± 10 °C/8 h/air (Figure 1).

**Figure 2:**
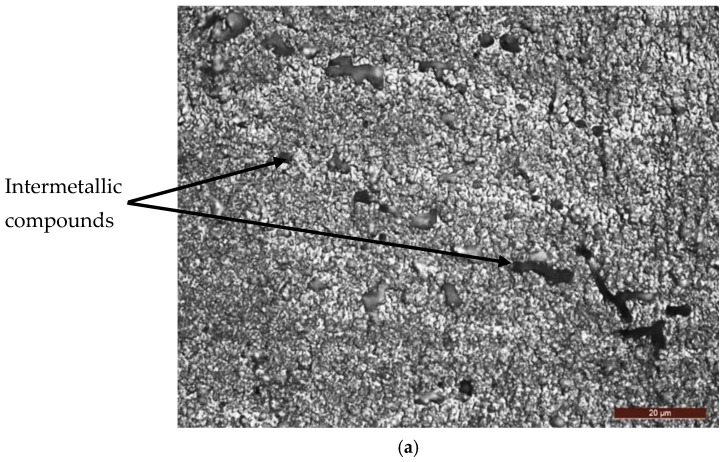

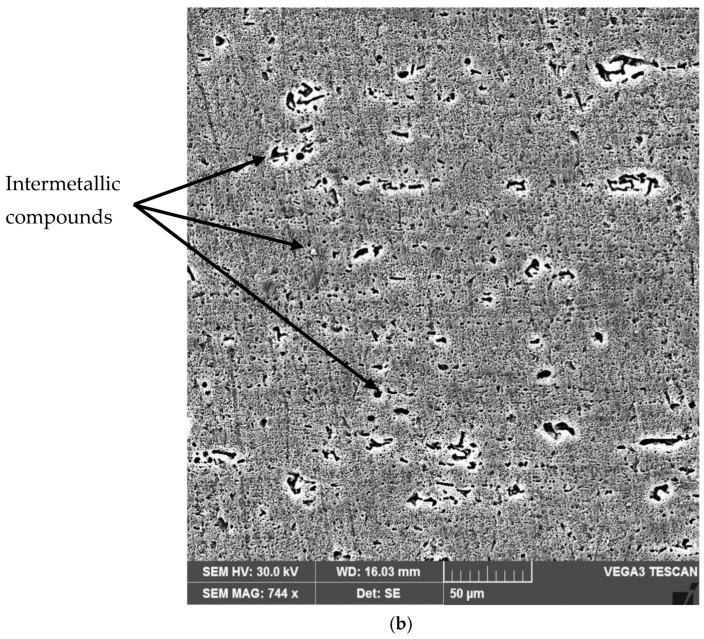
Microstructure of the alloy after solution heat treatment followed by artificial aging: (**a**) MO ×500; (**b**) SEM ×744.

Figure 2, the microstructure obtained after this heat treatment was composed of γ solid solution grains with an aluminum base having a polyhedral shape, and inside and on the separation boundaries between them, there were particles of intermetallic phases, consisting of Mg_2_Si for the large ones, Al_5_FeSi for those in the form of a plate, and Al_12_(Fe, Mn)_3_Si for the spherical ones [18]. This alloy shows the highest values for the mechanical resistance characteristics among all the 6xxx series alloys and is used in the aeronautical, marine, automotive and food industries [7,18].

**Figure 3:**
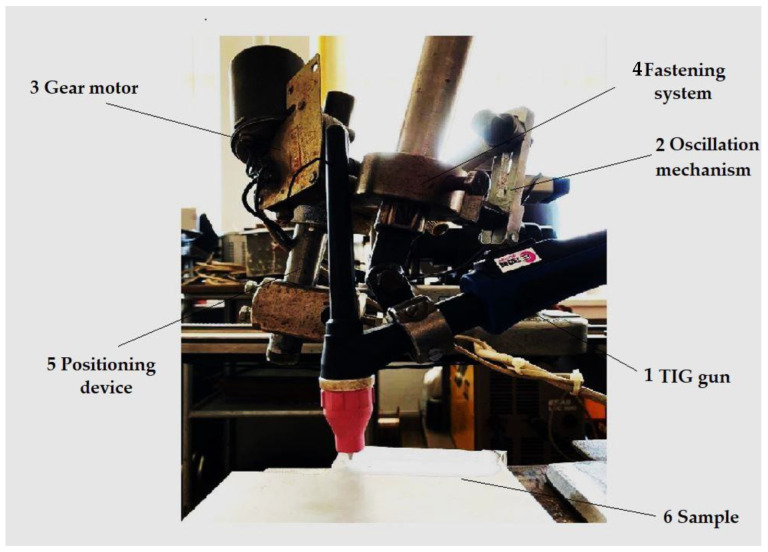
The welding experimental stand.

The surface was remelted using a TIG welding equipment (Weld Guru, Buda, TX, USA, Figure 3) in the following working conditions:

- base metal: Al-6082 alloy;
- welding equipment: MAGIC WAVE 300 (Fronius);
- alternating current frequency: 70 Hz;
- nature of current: alternating current;
- balance: 60/40;
- shielding gas: Ar 100%;
- electrode type: EWLa 15;
- electrode diameter: 3.2 mm;
- gas flow: 10 L/min;
- tilt of the gun: 90 degrees.

**Figure 4:**
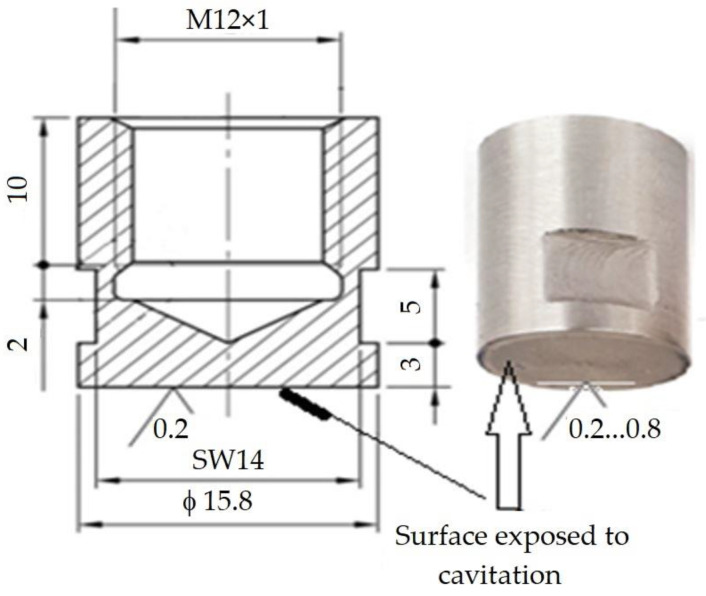
Geometry of the cavitation samples (mm).

Further, both from the locally surface remelted plates and from the reference ones, cavitation samples were processed by chipping; their shape and dimensions are shown in Figure 4.

The experimental research followed the study of the linear welding energy influence on the improvement of the cavitation erosion resistance of remelted surfaces. For that, three different welding regimes were used. A change in linear energy E_l_ = (U_a_ × I_s_)/v_s_ × 60 [J/cm] was achieved by changing the value of the welding current while keeping the welding speed constant, as follows:

- Regime 1:
  i. Welding current: Is = 100 A;
  ii. Electric arc voltage: Ua = 11 V (RMS = Root Mean Square);
  iii. Arc length: 2 mm;
  iv. Welding speed: vs. = 10 cm/min;
  v. Linear energy: E_l_ = 6600 J/cm.
- Regime 2:
  i. Welding current: Is = 150 A;
  ii. Electric arc voltage: Ua = 12.1 V (RMS);
  iii. Arc length: 2 mm;
  iv. Welding speed: vs. = 10 cm/min;
  v. Linear energy: E_l_ = 10,890 J/cm.
- Regime 3:
  i. Welding current: Is = 200 A;
  ii. Electric arc voltage: Ua = 13.2 V (RMS);
  iii. Arc length: 2 mm;
  iv. Welding speed: vs. = 10 cm/min;
  v. Linear energy: E_l_ = 15,840 J/cm.

**Figure 5:**
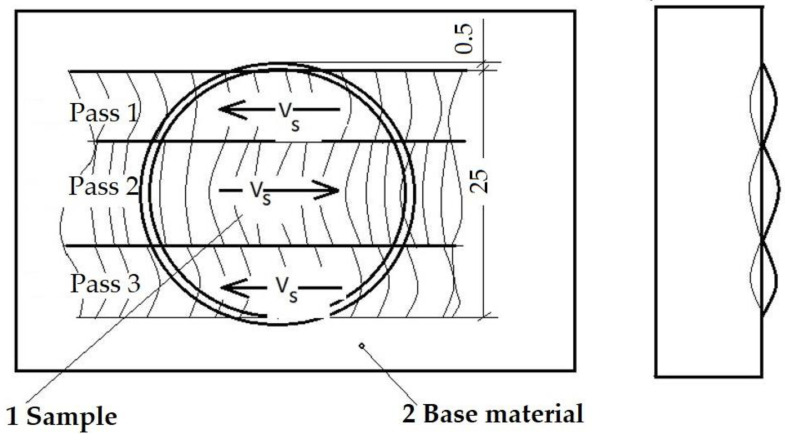
Details of the surface TIG remelting process.

According to the technological recommendations, the base material was preheated to a temperature of 100 °C, and the temperature between passes was maintained at values of 120–150 °C. In order to maintain a constant welding speed, the TIG welding head was mounted on a welding installation, the process being mechanized. For the surface remelting of the samples, parallel passages were made, with a step between passages equal to 2/3 of the width of a passage so as to achieve an overlap of the passages of approx. 1/3 of the width of a passage. The remelting details are shown in Figure 5. This made it possible to obtain a smooth molten surface without welding defects (lack of melting or marginal notches). The passages performed aimed at obtaining a width of the remelted area of at least 25 mm to provide cavitation samples with well-specified dimensions, shown in Figure 3.

**Figure 6:**
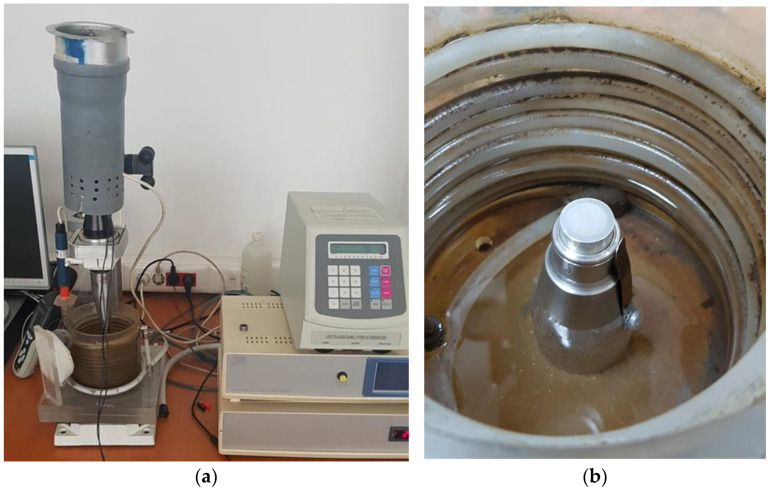
Experimental stand: (**a**) overview; (**b**) detail of the sample-fixing system.

The cavitation tests were conducted on sets of three samples for each remelting regime, using a vibrating device with piezoceramic crystals (Figure 6) [7,8] made in accordance with the requirements of Standard ASTM G32-2016 regarding the indirect testing method [19].

The functional parameters of the device were:

- vibration amplitude, 20,000 ± 1% Hz;
- vibration amplitude, 50 µm;
- power of the electronic ultrasound generator, 500 W;
- working environment, potable water having a temperature of 22 ± 1 °C.

A characteristic of this device is the control and constant maintenance of the acoustic and electrical parameters, with the help of a computer, based on a software implemented for this purpose.

Before the cavitation test, the attack surface of each sample was polished on a Buehler Phoenix Beta machine (Spectrographic Ltd., Shipley, UK) to a roughness Ra = 0.2 ÷ 0.8 µm. The total testing duration of each sample was 165 min, this being divided into 12 periods (one of 5 min, one of 10 min, and the next 10 periods of 15 min each). At the end of each test period, the sample was cleaned in acetone and air-dried.

Prior to the start of the tests and at the end of each intermediate test period, the cavitation exposed surfaces were examined under an optical Olympus SYX7 (Olympus, Zhengzhou, China) microscope and photographed with a high-resolution Canon Power Shot A 480 camera (Canon, Tokyo, Japan) to monitor the surface damage exposed to the cavitation attack. The samples were weighed before starting the tests and at the end of each intermediate period. The weighing was carried out with an analytical Zatklady Mechaniki Precyzyjnej WP 1 (Mechaniki Precyzyjnej R&G S.A., Mielec, Poland) balance, whose accuracy was of 5 significant decimals (up to 0.00001 g).

At the end of each intermediate test period, “*i*”, the corresponding mass loss was determined, Δ*m_i_*.

The eroded mass was established according to the relation:

(1) $$m_{i}=\sum_{i=1}^{12}\Delta m_{i}$$

Later, based on the mass losses, the values of the cumulative mean penetration depth of erosion, *MDE*_Σ*i*_ and the values of the erosion penetration rate related to the period “*i*” *MDER_i_* were determined [20,21]:

(2) $${\mathrm{MDE}}_{\Sigma i}=\sum_{i=1}^{12}\Delta MDE_{i}\;=\;\sum_{i=1}^{i=12}\frac{4\;\Delta m_{i}}{\rho \;\pi \;d_{p}^{2}}\;[µm]$$

*MDER_i_* = Δ*MDE_i_*/Δ*t_i_* [µm/min] (3)

where:

*i* is the testing period;

Δ*m_i_* is the mass of material lost through erosion, in period *i*, in grams;

*Ρ* is the material density, in grams/mm^3^;

Δ*t_i_* is the duration of cavitation corresponding to period “*i*” (5 min, 10 min or 15 min);

*d_p_* is the diameter of the sample surface subjected to cavitation attack (*d_p_* = 15.8 mm);

Δ*MDE_i_* is the value of the mean penetration depth of erosion, achieved by cavitation during the period Δ*t_i_*_._

At the end of the test, the cavitation exposed surface of the samples was examined by an optical Leica DM 2700 M microscope (Leica Microsystems, Madrid, Spain) and by the scanning electron microscope TESCAN VEGA 3 LMU Bruker EDX Quantax (Bruker Corporation, Billerica, MA, USA).

## 3. Results and Discussion

### 3.1. Cavitation Erosion Curves

**Figure 7:**
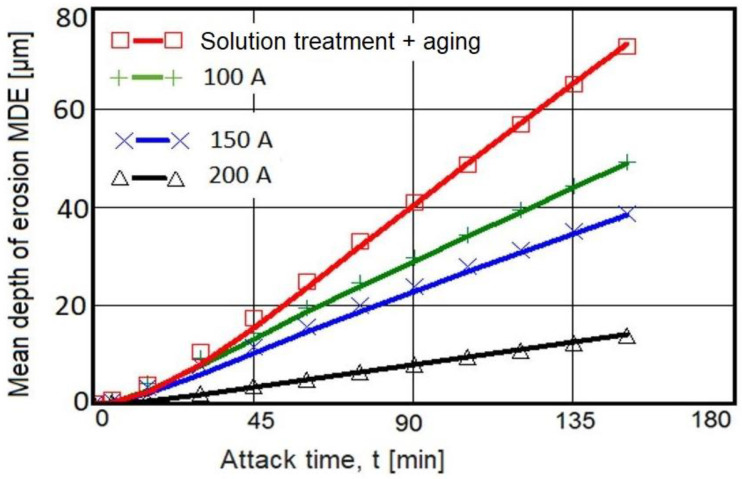
Evolution of the mean penetration depth of erosion with the cavitation attack time.

**Figure 8:**
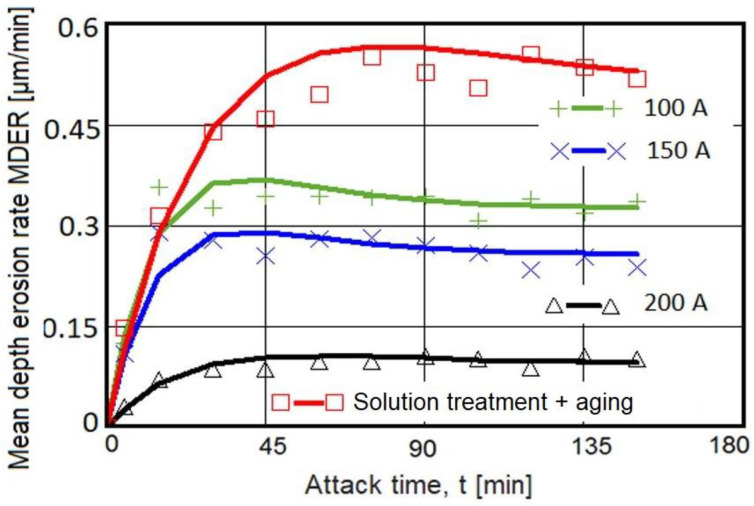
Evolution of the mean erosion rate with the cavitation attack time.

In Figure 7 and Figure 8, the characteristics of the cavitation erosion curves are shown comparatively and indicate the variation of the parameters MDE (mean penetration depth of erosion) and MDER (mean penetration rate of erosion) with the duration of the vibrating cavitation attack, for the three structural states obtained after local surface remelting as well as for the reference material subjected to the solution-based heat treatment followed by artificial aging. It is specified that the experimental values in these diagrams represent the mean values of the three tested sets of samples, for each type of material processing.

The following observations resulted from the analysis of these graphs:

- TIG remelting of the considered alloy surface at the currents Is = 100 A, 150 A, and 200 A caused an increase in the cavitation erosion resistance, Rcav, from 1.5 times to 5 times (where Rcav. = 1/MDER, t = 165), compared to the conventional heat treatment specific for this material;
- the use of welding currents of 200 A provided the lowest values of the MDE and MDER;
- during the stabilization period, until the end of the test duration (165 min), the erosion rates of the remelted TIG surfaces acquired values of approx. 0.10–0.32 µm/min, and those specific to the age hardening heat treatment were approx. 0.50 µm/min.

### 3.2. Macro- and Micrographic Examinations

#### 3.2.1. Macrographs of the Surfaces Tested for Cavitation

**Figure 9:**
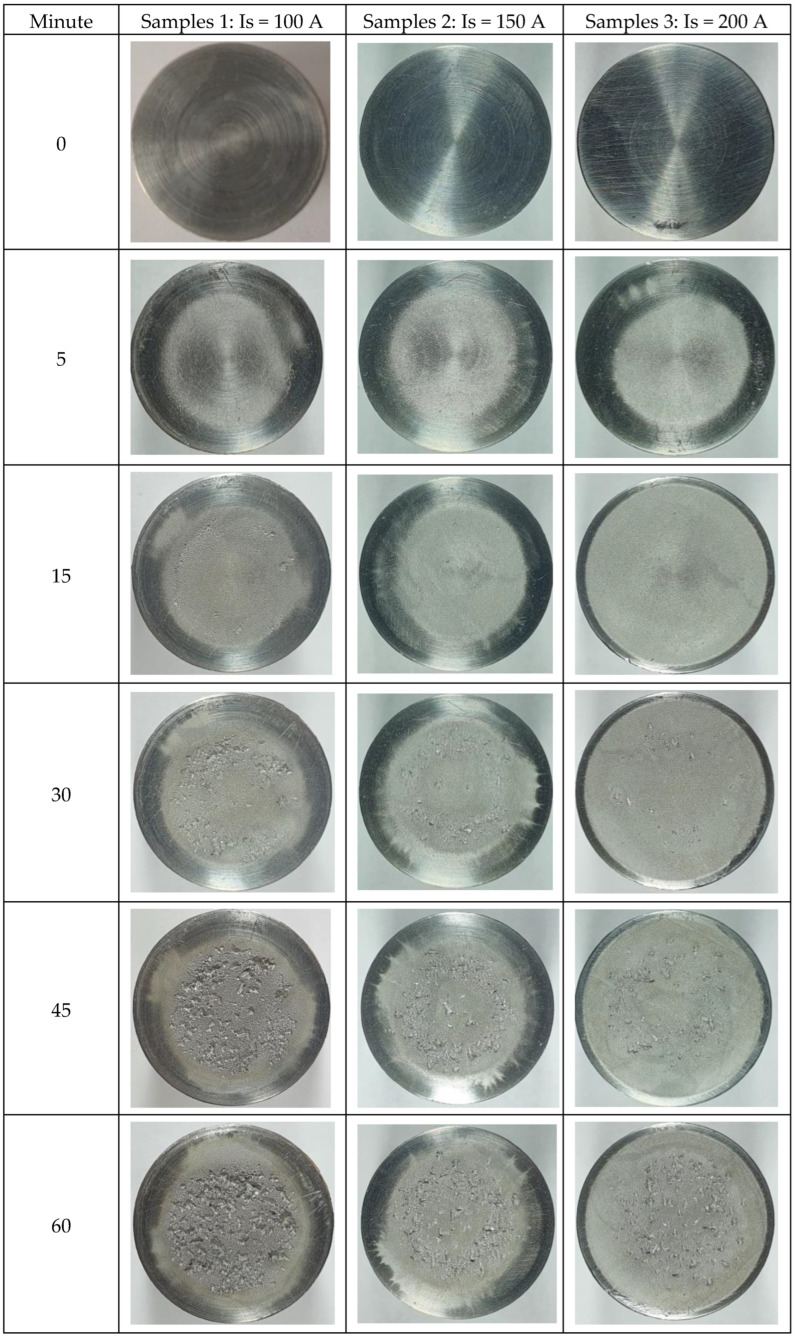

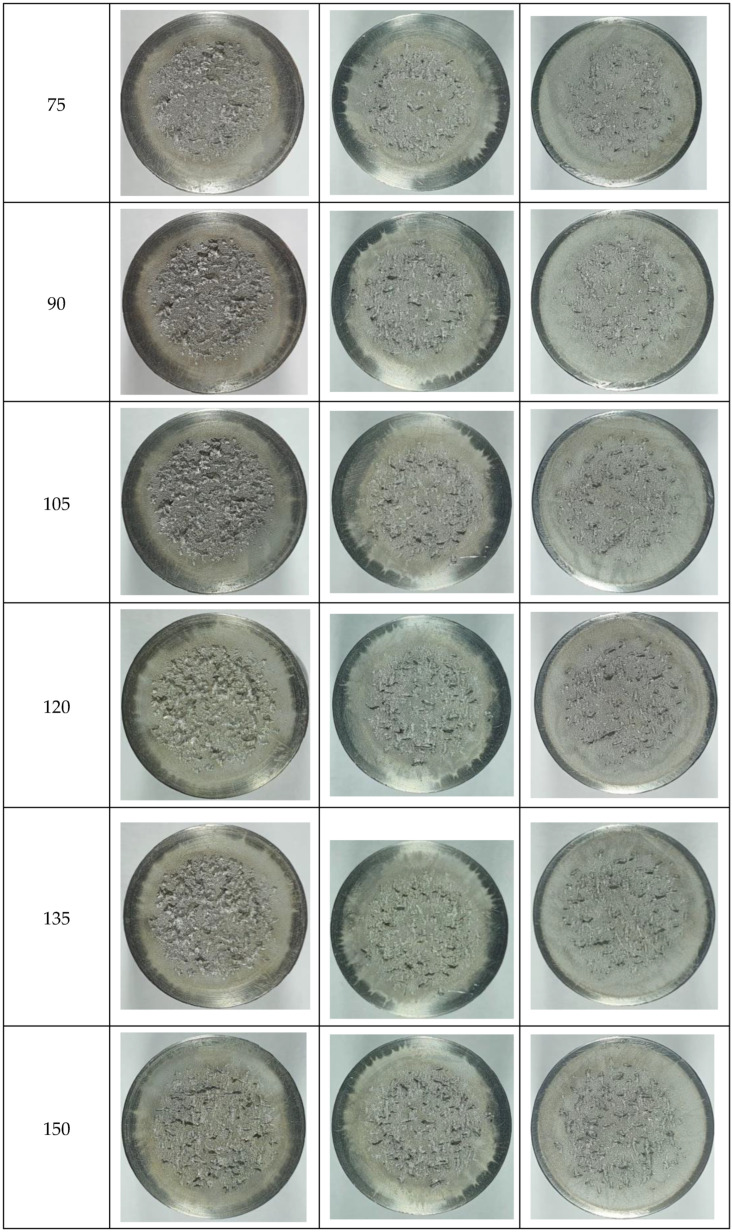

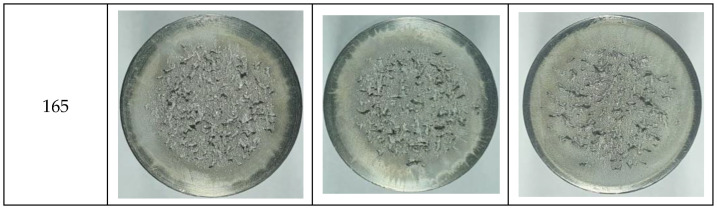
Macrographic images of the surface in cavitation tests of variable durations.

With the help of a Canon Power Shot A480 camera, images of the cavitation exposed surfaces were obtained at each test time. Figure 9 shows these characteristic images for each value of melting current with respect to the linear energy. The energetic impact generated by the collapse of the cavitation bubbles made the samples surface uneven. Up to 30 min of attack, no surface degradation was observed. At longer times, the formation of a ring from the surface periphery together with more and more microcraters (pinches) with variable sizes and uneven distribution was revealed. Thus, for cavitation attack times greater than 90 min, the remelted samples surfaces, especially with Is = 100 A and Is = 150 A, became very rough, which implies a strong damage to them. On the contrary, the degree of surface damage of samples remelted with Is = 200 A was lower, which proved that their cavitation erosion resistance was significantly improved. The main reason for this phenomenon can be the existence of fine grains and a microstructure with a uniform distribution of the constituent phases. After cavitation erosion for 165 min, the pinches on the material surface developed into an irregular group of cavitation craters.

#### 3.2.2. Micrograph of the TIG Remelted Layer

**Figure 10:**
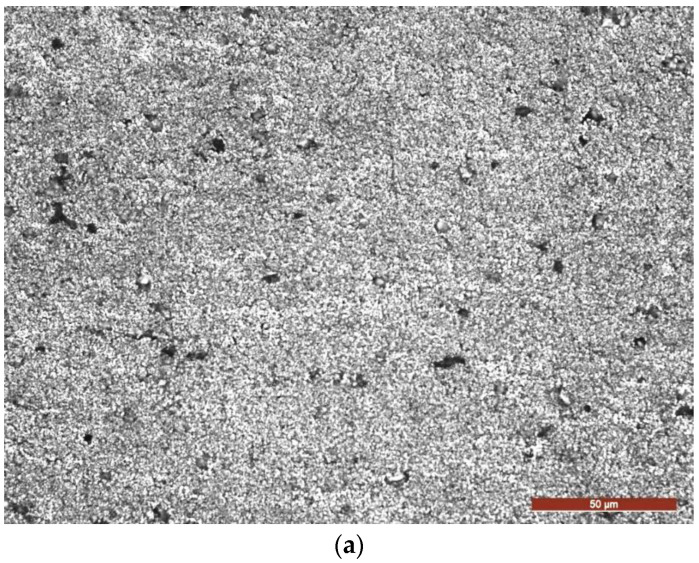

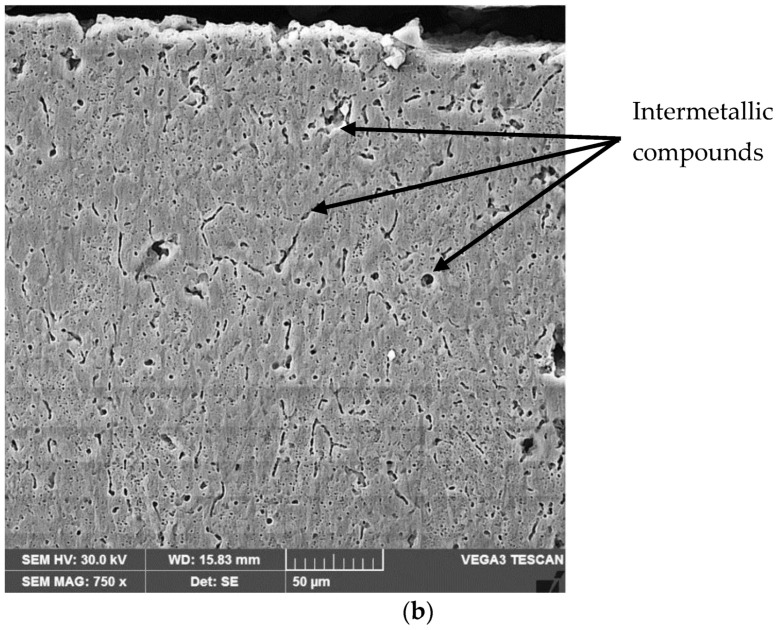
Microscopic images of a cross section through the remelted layer, with Is = 200 A: (**a**) OM ×200; (**b**) SEM ×750.

After the TIG remelting process, the surface of the material solidified at a higher degree of subcooling, with respect to that at a lower real temperature than the equilibrium temperature, so that the dimensions of the germs were smaller, their number was larger, and the microstructure obtained was finer. In Figure 10a,b the microstructure of the layer obtained at a current I = 200 A and a linear energy E_l_ = 15,840 J/cm is shown. It was observed that it contained small, equiaxial, unoriented crystals of α solid solution with an aluminum base and fine particles of intermetallic compounds [18], a microstructure that provides isotropic mechanical properties and an improvement of the use characteristics due to the strengthening mechanism resulting from the refinement of the granulation. The initiation and subsequent propagation of microcracks in the surface layer occurred at the interface between the particles derived from chemical combinations and the aluminum-based solid solution matrix (Figure 10b). Exposure of the material surface to repeated shock waves and/or repeated microjets led to an accumulation of plastic deformations until micro-damages occurred that caused material loss. With the increase of the cavitation attack time, there was a fragile removal of the phases of the chemical combinations, after which a uniform degradation of the solid solution grains took place, the breaking phenomenon having a ductile character.

**Figure 11:**
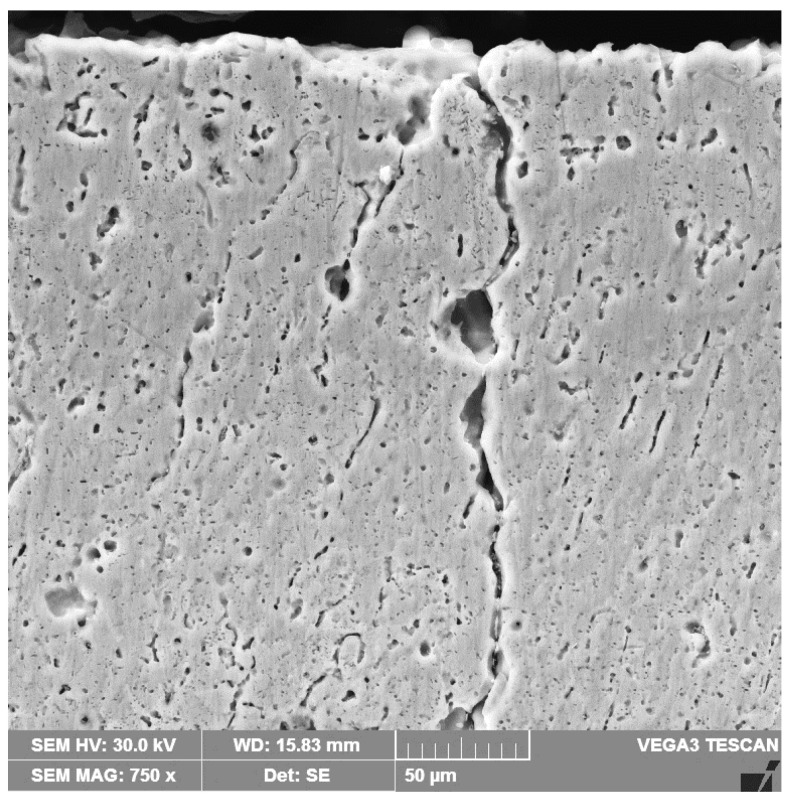
SEM image of a cross section with microcracks through the remelted layer, with Is = 250 A.

At melting current values over 200 A and linear energies higher than 15,840 J/cm, the very high thermal shock action induced by the surface melting process caused the appearance of microcracks in the edge layer (Figure 11).

The micro-hardness of the alloy subjected to heat treatment with a solution followed by artificial aging showed values of 112–120 HV 0.05, and that of the TIG remelted surfaces reached values of approx. 128–137 HV 0.05.

#### 3.2.3. Topography of Surfaces Eroded by Cavitation

**Figure 12:**
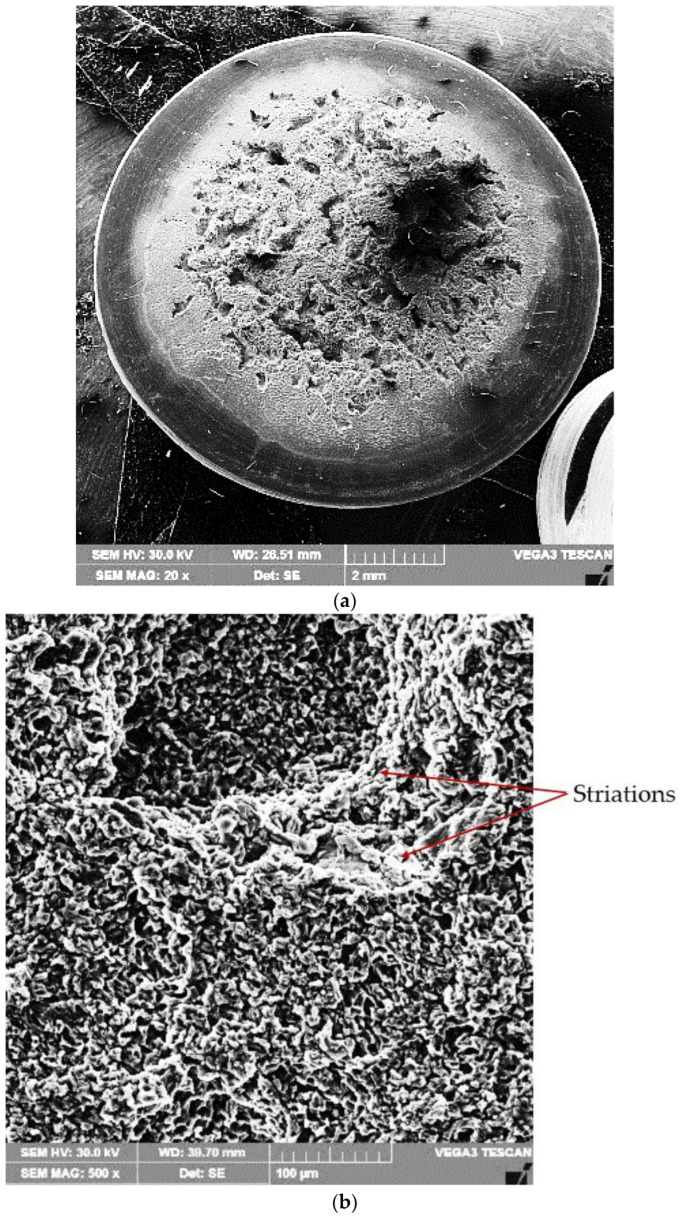

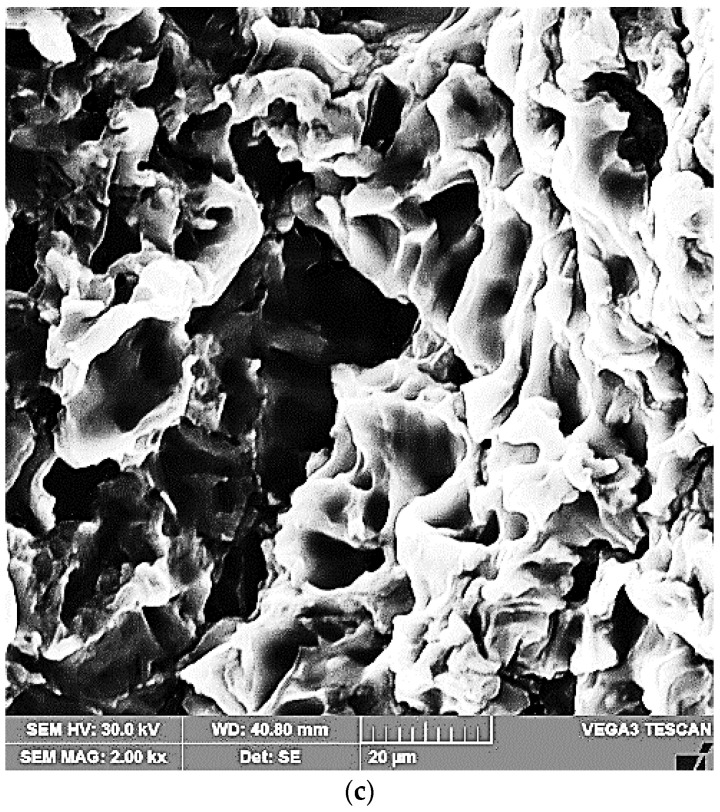
SEM image of the remelted surface, with Is = 100 A, and tested after 165 min for cavitation: (**a**) ×20; (**b**) ×500; (**c**) ×2000.

**Figure 13:**
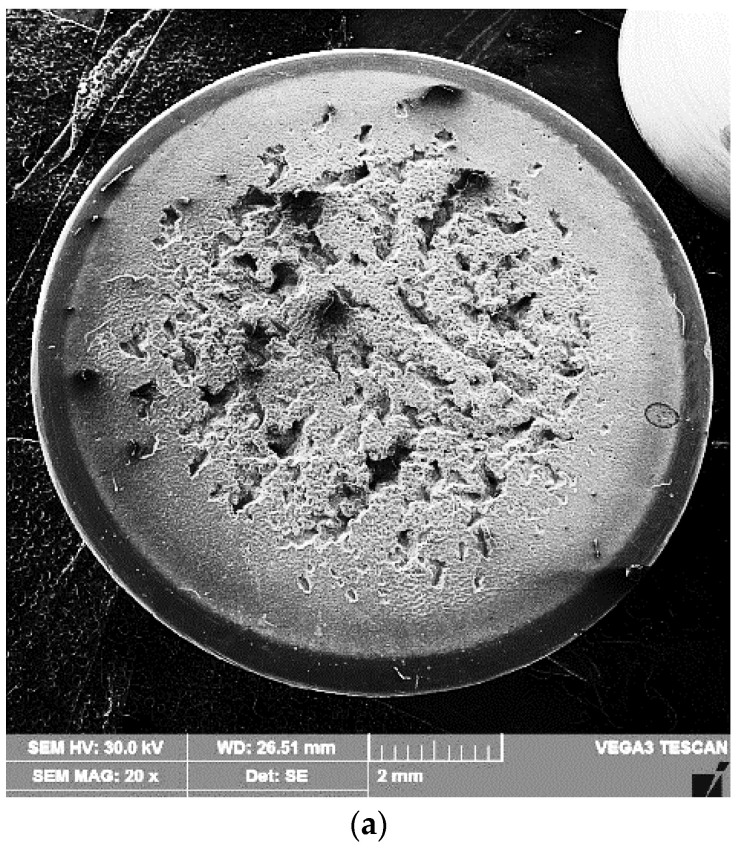

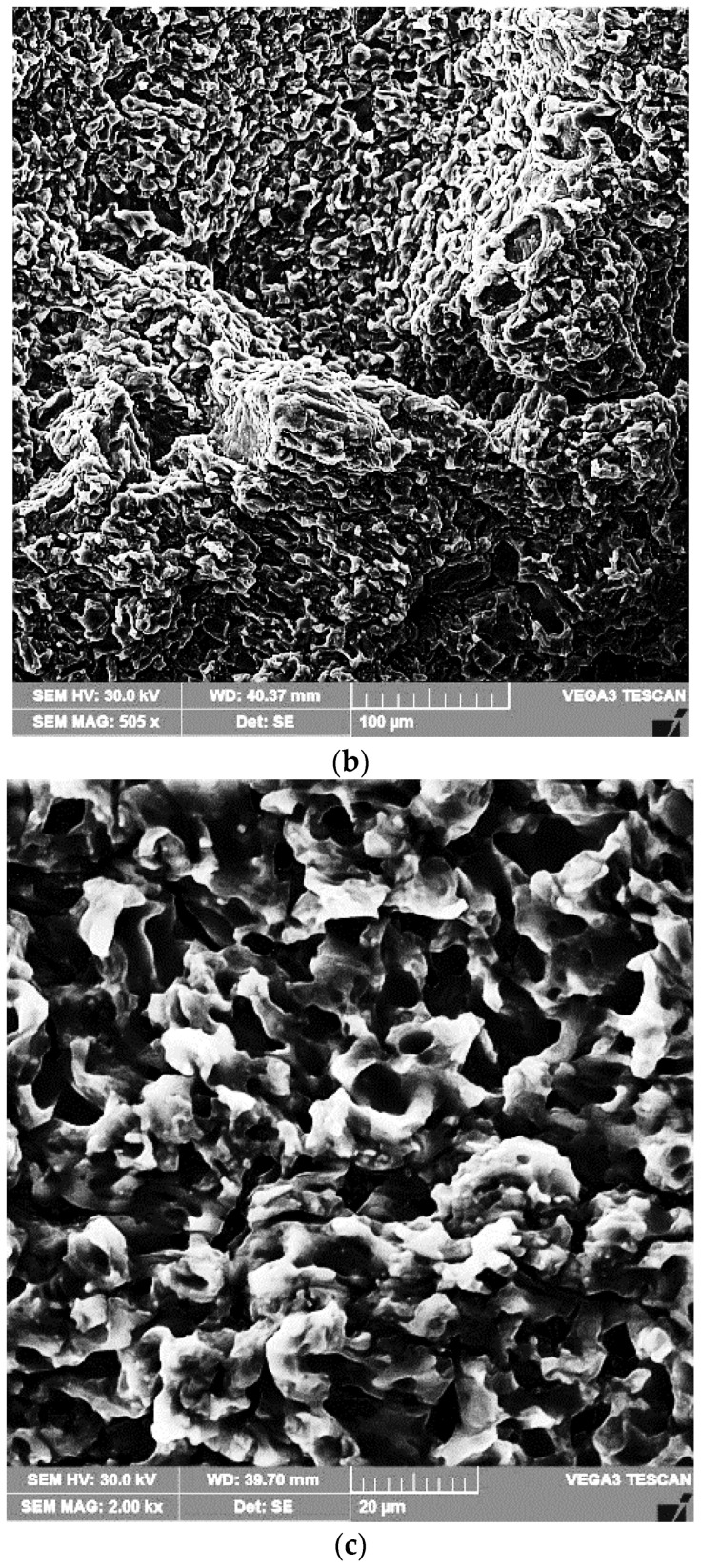
SEM image of the remelted surface, with Is = 200 A, and tested after 165 min for cavitation: (**a**) ×12: (**b**) ×500; (**c**) ×2000.

A scanning electron microscope examination of the surfaces tested for cavitation (Figure 12 and Figure 13) demonstrated that the erosion phenomenon occurred in a similar way to that of pure metals with a face-centered cubic lattice, fcc, namely, through plastic deformation followed by a ductile fracture over the entire surface.

The results of the metallographic investigations carried out with the optical microscope and the scanning electron microscope, in correlation with the hardness measurements, allowed explaining the mechanism of the cavitation erosion phenomenon. The finishing of granulation and microstructure after the local surface remelting process, beside the hardness increase, were responsible for the cavitation erosion improvement, along with the material removal. The high capacity of mechanical embrittlement (low stacking fault energy) of the solid solution matrix with the fcc crystalline network led to erosion by ductile fracture on the entire surface. The loss of material occurred preferentially in isolated regions, apparently with a random distribution, the formed microcraters showing typical striations similar to the fatigue fractures of aluminum alloys [4,6,9,13].

However, with this alloy containing up to 3% of alloying elements, the material was preferentially lost in isolated regions, apparently randomly, leaving behind striated, flat-bottomed microcraters [1,2,4,7]. The fracture surface of these microcraters was similar to that of fatigue fractures in aluminum alloys. The microcraters expanded laterally to macroscopic sizes without undergoing changes in their topographical characteristics. The diameter of the cavitation microcraters was approximately 20 μm, and some of them reached 50 μm and even 80–90 μm.

## 4. Conclusions

Based on the investigations regarding the microstructure and cavitation erosion characteristics of TIG remelted surfaces of the EN AW-6082 alloy, the following conclusions can be summarized:

The process of local surface remelting using the TIG electric arc, operated at currents of 100, 150, and 200 A and voltage of 10 V, led to grain and microstructure refinement in the investigated alloy.

It was highlighted that for welding current values Is = 200 A, the cavitation erosion resistance of this alloy increased by a factor of approximately 200%.

The surface microhardness of the structurally modified layers increased from approx. 115 HV0.05 to approx. 134 HV0.05.

The fracture surface of the microcraters formed on the hollow surfaces had a ductile character, similar to the fatigue fractures of aluminum alloys.

## Data Availability

Not applicable.
